# The effect of Cinnamaldehyde on mucositis and salivary antioxidant capacity in gamma-irradiated rats (a preliminary study)

**DOI:** 10.1186/2008-2231-20-89

**Published:** 2012-12-10

**Authors:** Tahereh Molania, Ali Akbar Moghadamnia, Mahdi Pouramir, Sara Aghel, Dariush Moslemi, Leila Ghassemi, Mina Motallebnejad

**Affiliations:** 1 Department of Oral Medicine, Dental School, Mazandaran University of Medical Sciences, Sari, Iran; 2 Department of Pharmacology, Babol University of Medical Sciences, Babol, Iran; 3 Department of Biochemistry, Babol University of Medical Sciences, Babol, Iran; 4 Department of Oral Medicine, Dental School, Babol University of Medical Sciences, Babol, Iran; 5 Shahid Rajaee Hospital, Babol University of Medical Sciences, Babol, Iran; 6 Cellular and Molecular Biology Research Center, Dental School, Babol University of Medical Sciences, Babol, Iran

**Keywords:** Cinnamaldehyde, Saliva, Mucositis, Antioxidant, Rat

## Abstract

**Background and purpose of the study:**

The aim of this study was to investigate the effect of cinnamaldehyde on mucositis and salivary total antioxidant capacity in gamma-irradiated rats.

**Methods:**

The study was conducted on 28 male Wistar rats, 7–11 weeks of age and 160 ± 20 g body weight, divided into four groups of seven rats each. The first group receiving normal saline (S), the second group receiving saline and gamma radiation (SR), the third group receiving 50 mg/kg cinnamaldehyde 98% (C), and the fourth group receiving 50 mg/kg cinnamaldehyde 98% and gamma radiation (CR). SR and CR groups were exposed to 15 Gy gamma irradiation for 7 min and 39 s. Rats were intraperitoneally injected each day during the 10-day period of the experiment, and their tongues and lips were examined to assess the incidence and severity of mucositis. The saliva samples were taken from the animals three times on day zero, six, and ten.

**Results:**

The mean mucositis incidence appeared to be delayed in the CR than the SR group (P = 0.024), and the severity was significantly higher in the SR compared to the CR group;the difference was statistically significant on the second day (P = 0.027). In the evaluation of salivary antioxidant capacity, salivary antioxidant concentration was significantly higher in the C than the S, SR, and CR groups on the tenth day of the experiment (p <0.05).

**Conclusion:**

The clinical effects in the CR group seem to be due to antioxidant, anti-bacterial and anti-inflammatory effects of cinnamaldehyde; this conclusion, however, requires further investigations. Delayed antioxidant effect in the CR group was evident in ip cinnamaldehyde injection, the exact mechanism is not clear.

## Background

Oral mucositis is the most common and distressing side effect of cancer chemotherapy and radiotherapy. Almost every patient with oral cancer treated with chemo-radiotherapy develops deep and painful wounds as the characteristic of this condition [1]. The wounds often affect the gastrointestinal mucosa, and mucositis can, therefore, results in severe discomfort and reduction in patient's ability to eat, swallow and speak [2]. Radiation therapy leads to the production of free radicals and subsequent oxidative stress, thereby causing damage to cells and their function. It seems that radiotherapy-induced oxidative stress can also affect the salivary antioxidant capacity [3].

*Cinnamon*, scientifically named *Cinnamomum spp*, is a plant with many uses as a herbal medicine, containing mucilage, tannin, sugar, resin, and essential oil, among which the essential oil is the most important part, a substantial portion of which is made up of cinnamaldehyde or cinnamic aldehyde [4]. In traditional medicine, various therapeutic uses have been proposed for this plant [5-7]; besides, it has been found to have high antioxidant [8], anti-bacterial [9], and anti-inflammatory [10] activity which also play a role in tissue repair [11]. Various agents have been studied for the management or prevention of mucositis, and the recent evidences suggest that cryotherapy, benzydamine, corticosteroid [2], GM-CSF [12], amifostine [2,13] and palifermin [2,14] may be helpful in certain situations via blood vessels contraction, anti-inflammatory and local anesthetic effects, anti-inflammatory properties, and cytoprotective mechanism respectively. Therefore, regarding the pharmacologic effects of cinnamaldehyde, the present study aimed at investigating the antioxidant property of cinnamaldehyde on the rats' saliva and its clinical effects on mucositis.

## Methods

The present experimental study was carried out on 28 male albino Wistar rats,(7 to 11& 160 ± 20 g), selected through simple random sampling from the Animal Care Center of Babol University of Medical Sciences. The project was approved by the Research Council as well as the Research Ethics Committee of Babol University of Medical Sciences (ORN: 1050). Prior to the study, all animals were kept under the same laboratory condition at 21 ± 1°C and 12:12 h light/dark cycle with same access to food and water supply [15]. Cinnamaldehyde 98% (Merk, Germany) was used in the present study. Rats were divided into four groups of seven rats each, The first group receiving normal saline (S), the second group receiving saline and gamma radiation (SR), the third group receiving 50 mg/kg cinnamaldehyde 98% (C), and the fourth group receiving 50 mg/kg cinnamaldehyde 98% and gamma radiation (CR).

A pilot study was conducted to observe the process of radiation, radiation induced-mucositis onset, determination of the maximum radiation effects (sixth day) and recovery from mucositis (tenth day). Prior to the radiation, all rats were marked and weighed. Normal saline was then injected to S and SR groups and cinnamaldehyde to C and CR groups as the first intraperitoneal(ip) injection. On the same day, animals were partially anesthetized with midazolam (25 mg/kg), and pilocarpine 0.5 mg/kg was injected ip to obtain the saliva samples. Fifteen minutes later, saliva samples were taken using a sampler and collected in 0.5 ml microtubes. In the afternoon, the CR and SR groups were transferred to Shahid Rajai hospital of Babolsar for receiving the radiation. The animals were anesthetized by ketamine (100 mg/kg) ip before the radiation; they were completely immobilized on a special shield and exposed dosage 15 Gy gamma radiation (16Co and 1.25 million electron volts energy using Teraton780) at a for 7 min and 39 s. The tube placement was set in the way that the rats' whole cranium was in the field [15]. At the end of radiation, rats were returned to the Animal Care Center of Babol University of Medical Sciences and were daily weighted during the 10-day experimental period; the relative groups were intraperitoneal injected afterwards and their tongues and lips were examined for the signs of mucositis using Parkin's clinical scale [16] (scale 0, normal; scale 0.5, slightly pink; scale 2, extremely red; scale 3, local desquamation; scale 4, exudation and crust less than one-half of the lip; scale 5, exudation and crust more than one-half of the lip). On the sixth day of the experiment, as the mucositis peak (according to the pilot study), saliva samples were taken using the mentioned approach. On the last day of the study (the 10th day), improvement was evident in mucositis and radiation-induced effects. Saliva samples were collected one more time on this day, and the animals were sacrificed after anesthesia.

### Salivary analysis

The saliva samples prepared on days zero, six and ten, kept at −20°C in the freezer, were transported to the laboratory and underwent centrifugation at 3000 rpm for 15 min and supernatant transferred to the test tubes after deposition of saliva impurities, and the total antioxidant activity was calculated using the FRAP (Ferric reducing antioxidant power) technique [17]. In this method the reduction of Fe^3+^ to Fe^2+^ is seen in the presence of antioxidants. In sum, FRAP reagent contains TPTZ (2, 4, 6-tripyridyl-s-triazine; sigma) 10 mmol/L in 40 mmol/L HCL plus FeCl_3_ 20 mmol/L and buffer acetate 0.3 mol/L (PH: 3.6) in the ratio of 10:1:1; the reagent was freshly prepared and heated for 5 min at 37°C. The working FRAP reagent (1.5 ml) was mixed with 50 microliter of serum. After 10 min at 37°C, the absorption was read at 593 nm and compared with the standard. FeSo4 (125, 250, 500, and 1000 μmol/L) was considered as the standard solution, based on which the standard curve was plotted [18].

### Statistical analysis

Data are presented as the mean (±SD) in the tables and figures. For weight comparison, one-way ANOVA was used along with post-hoc Bonferroni. Mucositis-related data were also analyzed by one-way ANOVA. To compare the severity of mucositis between the treatment groups, Mann–Whitney was applied between each two groups. Data related to the salivary antioxidant capacity were analyzed by one-way ANOVA as well. P-value <0.05 was considered significant.

## Results

The present study was conducted on 28 Wistar rats,(7–11, 160 ± 20 g) divided into four groups of seven rats each. All the animals were alive during the ten-day study period and received the relative daily injections.

### Mucositis

The mean mucositis onset was 2.43 ± 0.2 days in the SR and 4.14 ± 1.6 days in the CR groups, so as mucositis appeared significantly later in CR than the SR group (P = 0.024, *T*-test).

**Figure 1:**
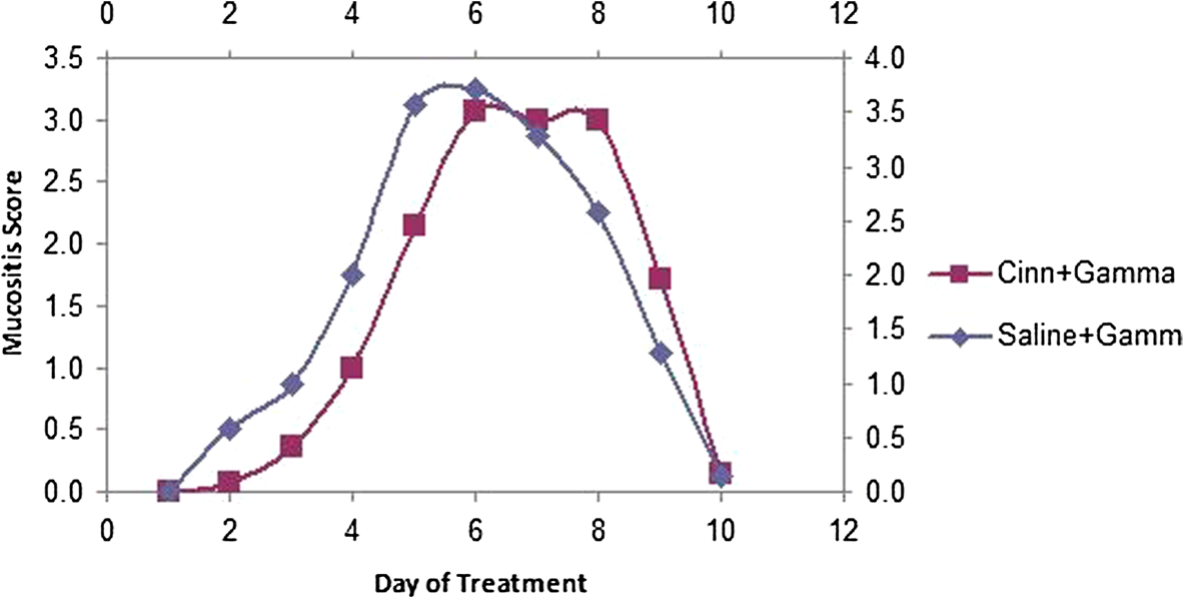
Alteration of mucositis in the CR and SR groups during the days of experiment.

The results revealed that the scale of mucositis was higher in SR compared to the CR group, and the difference was significant on the second day of the experiment (Mann–Whitney test, p = 0.027) (Figure 1, Table 1).

**Table 1:** The mean (±SD) scale of mucositis in SR and CR groups in the days of experiment

**Days**	**0**	**1**	**2**	**3**	**4**	**5**	**6**	**7**	**8**	**9**	**10**
**Mucositis**											
SR	0.00	0.00	0.56 ± 0.44	1.00 ± 0.76	2.00 ± 0.81	3.57 ± 0.44	3.71 ± 1.11	3.28 ± 0.95	2.57 ± 1.27	1.28 ± 0.95	0.14 ± 0.37
CR	0.00	0.00	*0.07 ± 0.18	0.35 ± 0.47	1.00 ± 1.00	1.14 ± 1.77	3.07 ± 1.69	3.00 ± 1.41	3.00 ± 1.41	1.71 ± 1.41	0.14 ± 0.37

*Mann–Whitney test, p = 0.027.

### Antioxidant

**Figure 2:**
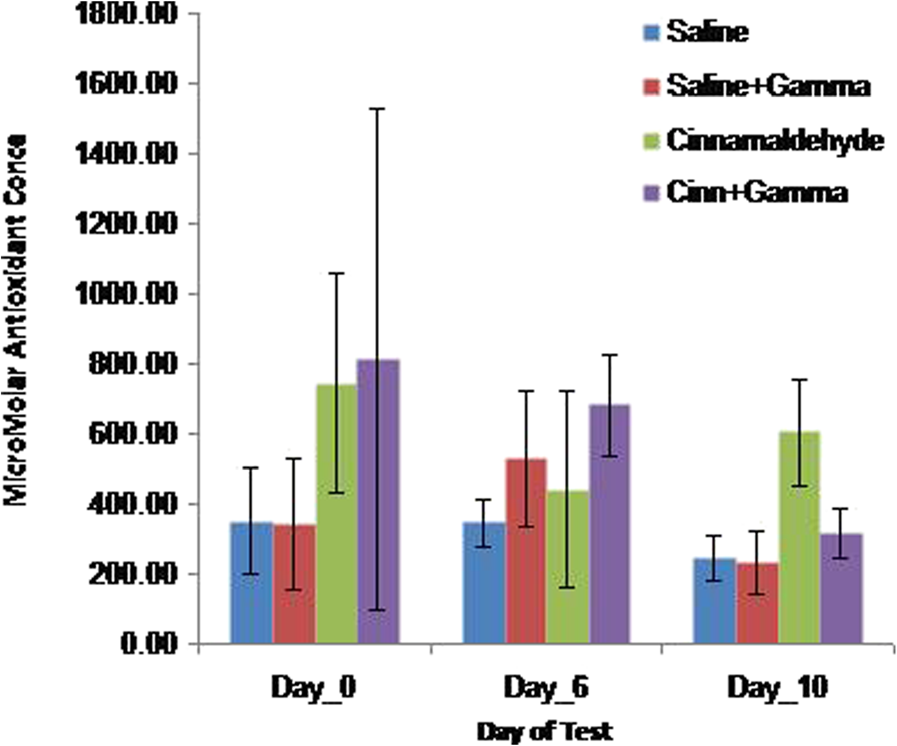
Total salivary antioxidant capacity (TAC) in the study groups.

Salivary antioxidants concentration showed no significant difference between the study groups on day zero (one-way ANOVA, p > 0.05). On the sixth day, the concentration of saliva antioxidant was significantly higher in CR compared to S group (one-way ANOVA, P = 0.017); however, salivary antioxidant concentration was remarkably higher in C compared to S, SR, and CR groups on the tenth day of experiment (one-way ANOVA, P <0.05) (Figure 2, Table 2).

**Table 2:** The mean (±SD) salivary antioxidant capacity (μM) in the four groups on days zero, six and ten

**Groups**	**CR**	**C**	**SR**	**S**
**Days**				
0	74/69 ± 813/50	315/23 ± 744/94	187/40 ± 344	29/151 ± 348/86
6	146/66 ± 681/79	280/40 ± 439/23	194/51 ± 527/5	17/67 ± 345/71
10	71/08 ± 606/23	150/73 ± 605/23	90/49 ± 230/94	62/90 ± 244/38

### Weight

The mean (±SD) weight change was 25.71 ± 9.53, -4.7 ± 31.1, -1 ± 11.4, and 11.42 ± 7.27 in the S, SR, C, and CR groups respectively. There was a significant weight gain in S compared to the C group (*T*-test, p = 0.042); similarly, the CR group showed a higher weight gain than the SR (*T*-test, P = 0.338), as well as the C group (*T*-test,p = 0.56).

## Discussion

In the present research, the effect of cinnamaldehyde has been investigated on mucositis and total salivary antioxidant capacity (TAC) in gamma-irradiated rats. Several studies have addressed to evaluate the anti-inflammatory, antibacterial and antioxidant effects of cinnamaldehyde. Reduction in the production of prostaglandin E2 and inhibition of cyclooxygenase-2 (COX-2) expression and subsequent significant decrease in Interleukin-1ß [19], increase in glutathione peroxidase activity [20], and bactericidal activity against *Streptococcus*[21] and *Staphylococcus* species, *lactobacilli*[17], and other gram positive and negative bacteria [22] are the results brought about as the effect of cinnamaldehyde. Clinical findings of the present study showed a significantly delayed onset of mucositis in CR than the SR group, and in terms of the mucositis severity, the difference of scale of mucositis was statistically significant on the second day of the experiment in the CR group. The clinical anti-mucositis effects were evident in CR compared to the SR group. Mucositis begins with inflammatory phase and continues with ulcerative and bacteriological phase [14]; On the other hand, radiotherapy leads to the induction of oxidative stress, thereby resulting in tissue damage [3]; thus, it is likely that through the anti-inflammatory and antioxidant mechanisms, cinnamaldehyde may delay the onset of mucositis. Moreover, alteration in oral microflora including the appearance of *Streptococcus mutans*, *lactobacilli* and gram-negative bacilli in the bacteriological phase can aggravate the severity of mucositis [14], and it seems that cinnamaldehyde can cause clinical influence on mucositis via its antibacterial properties; this conclusion, however, requires further and more accurate investigation. In addition, study on a greater number of samples in each group can perhaps describe the difference in the severity of mucositis between the two groups more precisely on more days during the ten-day period.

In a study by Gowder et al., it has been shown oral administration of cinnamaldehyde has a delayed effect on serum antioxidant of the rat's kidney tissue following passing through the liver metabolism, so as it contributes to an increase in antioxidants such as superoxide desmotase, glutathione proxidase, and glutathion-s-transferase [20]. The results achieved from the analysis of salivary antioxidant capacity indicated a significantly higher level of TAC in C compared to the other groups only on the tenth day of the experiment. It appears that ip injection of cinnamaldehyde may have a delayed effect on salivary TAC, the exact mechanism of which is not yet clear. Therefore, it is recommended that to shed light on the relative mechanism, studies be conducted in a longer period of time; the prophylactic use of cinnamaldehyde is also suggested to be initiated several days before the radiation and the antioxidant effects be evaluated during irradiation to benefit from antioxidant effects of cinnamaldehyde on mucositis improvement, since there are bodies of evidence indicating the delayed and time-dependent properties of cinnamaldehyde [20].

TAC demonstrated a decreasing trend in the CR group during the ten-day study period; nonetheless, it showed an increase compared to the SR group in mutual comparison between the study groups, as salivary antioxidant concentration was higher in CR than the SR group although the difference was not statistically significant. Regarding the radiotherapy-induced oxidative stress which may lead to DNA damage and loss of acinar precursors of the salivary glands [3], reduced TAC in the CR group might be due to damage to the salivary glands [3], as well as delayed and time-dependent effect of cinnamaldehyde [20]. Although, it is not clear that the oxidative stress induced by ionizing radiation on malignant cells may be faded by the anti-oxidative effects of cinnamaldehyde or any other remedies which have been suggested to be protective for normal tissues [23].

In the examination of weight change, a significant increase was observed in S compared to the C group; such a weight loss in the latter can be ascribed to the allergic and toxic nature of cinnamaldehyde [24] in comparison with the safe injection of normal saline. The weight change has not been statistically significant in the other groups.

In the end, it is suggested that further researches be implemented to assess the clinical and histopathological effect of cinnamaldehyde on radiotherapy-induced mucositis and the relative mechanisms.

## Conclusion

The clinical effects in the CR group seem to be due to antioxidant, anti-bacterial and anti-inflammatory effects of cinnamaldehyde; this conclusion, however, requires further investigations. Delayed antioxidant effect in the CR group was evident in ip cinnamaldehyde injection, the exact mechanism is not clear.

## Competing interests

The authors declare that they have no competing interest.

## Authors’ contributions

TM carried out acquisition of data, drafting the manuscript, AAM participated in pharmacologic process, interpretation of data and analysis, concept and design. MP carried out Biochemistry process, SA carried out acquisition of data. DM participated in radiation process, revising the manuscript. LG participated in acquisition of data. MM participated in concept and design, drafting the article, revising for important intellectual content. All authors read and approved the final manuscript.

## 

This project was approved and financially supported by the Council of Research and Technology, Babol University of Medical Sciences and undertaken in Shahid Rajaee Hospital, Dental School, Pharmacology and Biochemistry Departments of Babol University of Medical Sciences.
